# Supplementary material for: Optimizing the conservation of migratory species over their full annual cycle
Source: Nat Commun. 2019 Apr 15;10:1754. doi: 10.1038/s41467-019-09723-8 (PMC6465267; doi:10.1038/s41467-019-09723-8)
Supplement: Supplementary file 1 — Supplementary Information [file 41467_2019_9723_MOESM1_ESM.pdf]

Optimizing the conservation of migratory species over their full annual cycle

Schuster et al.

Supplementary Information

Supplementary Tables

Land cover	Area available	Single Population		Spatial Clustering of Species Abundance	
		Intact habitat	Shared-use	Intact habitat	Shared-use
Cropland/Mosaic Cropland	2269	596	895	875	1295
Grassland	5555	2091	3139	2387	3769
Urban areas	205	14	171	53	186
Broadleaf Deciduous Forest	1994	1032	1385	1217	1611
Broadleaf Evergreen Forest	6921	3436	1606	3984	4064
Needleleaf Forest	4599	2882	2806	2792	3266
Mixed Forest	966	610	749	611	850
Mosaic Forest	934	381	368	431	527
Flooded Forest	540	287	230	312	369
Shrubland	4226	1763	1925	2169	2498
Wetland	468	219	161	287	276
Barren	1053	415	222	441	303
**Total**	**31615**	**13727**	**13656**	**15558**	**19015**

Supplementary Table 1. Area selected (1000 km^2) for major land cover types using weekly planning for shared-use vs. sparing scenarios and for single population vs spatial clustering approaches. Area available is the total amount of each land cover available based on all cells throughout the year where $\geq$ 1 species was present. Not all land cover classes are included in the table and therefore individual land cover values do not sum to the total in each column. Land cover data was extracted from the global land cover map for 2015 (300m resolution).

ESA Climate Change Initiative. Global land cover map 300m resolution for 2015. (2017). Available at: http://maps.elie.ucl.ac.be/CCI/viewer/download.php.

Summary class	Individual classes
Cropland/Mosaic Cropland	Cropland/rainfed
Cropland/Mosaic Cropland	Cropland, irrigated or post-flooding
Cropland/Mosaic Cropland	Mosaic cropland (>50%) / natural vegetation (tree, shrub, herbaceous cover) (<50%)
Cropland/Mosaic Cropland	Mosaic natural vegetation (tree, shrub, herbaceous cover) (>50%) / cropland (<50%)
Grassland	Herbaceous cover
Grassland	Grassland
Urban areas	Urban areas
Broadleaf Deciduous Forest	Tree cover, broadleaved, deciduous, closed to open (>15%)
Broadleaf Deciduous Forest	Tree cover, broadleaved, deciduous, closed (>40%) (61)
Broadleaf Deciduous Forest	Tree cover, broadleaved, deciduous, open (15-40%) (62)
Broadleaf Evergreen Forest	Tree cover, broadleaved, evergreen, closed to open (>15%)
Needleleaf Forest	Tree cover, needleleaved, evergreen, closed to open (>15%)
Needleleaf Forest	Tree cover, needleleaved, evergreen, closed (>40%) (71)
Needleleaf Forest	Tree cover, needleleaved, evergreen, open (15-40%) (72)
Needleleaf Forest	Tree cover, needleleaved, deciduous, closed to open (>15%)
Needleleaf Forest	Tree cover, needleleaved, deciduous, closed (>40%) (81)
Needleleaf Forest	Tree cover, needleleaved, deciduous, open (15-40%) (82)
Mixed Forest	Tree cover, mixed leaf type (broadleaved and needleleaved)
Mosaic Forest	Mosaic tree and shrub (>50%) / herbaceous cover (<50%)
Mosaic Forest	Mosaic herbaceous cover (>50%) / tree and shrub (<50%)
Flooded Forest	Tree cover, flooded, fresh or brakish water
Flooded Forest	Tree cover, flooded, saline water
Shrubland	Shrubland
Shrubland	Shrubland evergreen
Shrubland	Shrubland deciduous

Wetland	Shrub or herbaceous cover, flooded, fresh/saline/brackish water
Barren	Lichens and mosses
Barren	Sparse vegetation (tree, shrub, herbaceous cover) (<15%)
Barren	Bare areas
Barren	Consolidated bare areas
Barren	Unconsolidated bare areas

Supplementary Table 2. Summarized and individual land cover classes used to examine selection of land cover types under single season vs. full annual cycle planning and for shared-use vs sparing scenarios. See ref. 44 for further descriptions of the individual land cover classes.

Supplementary Figures

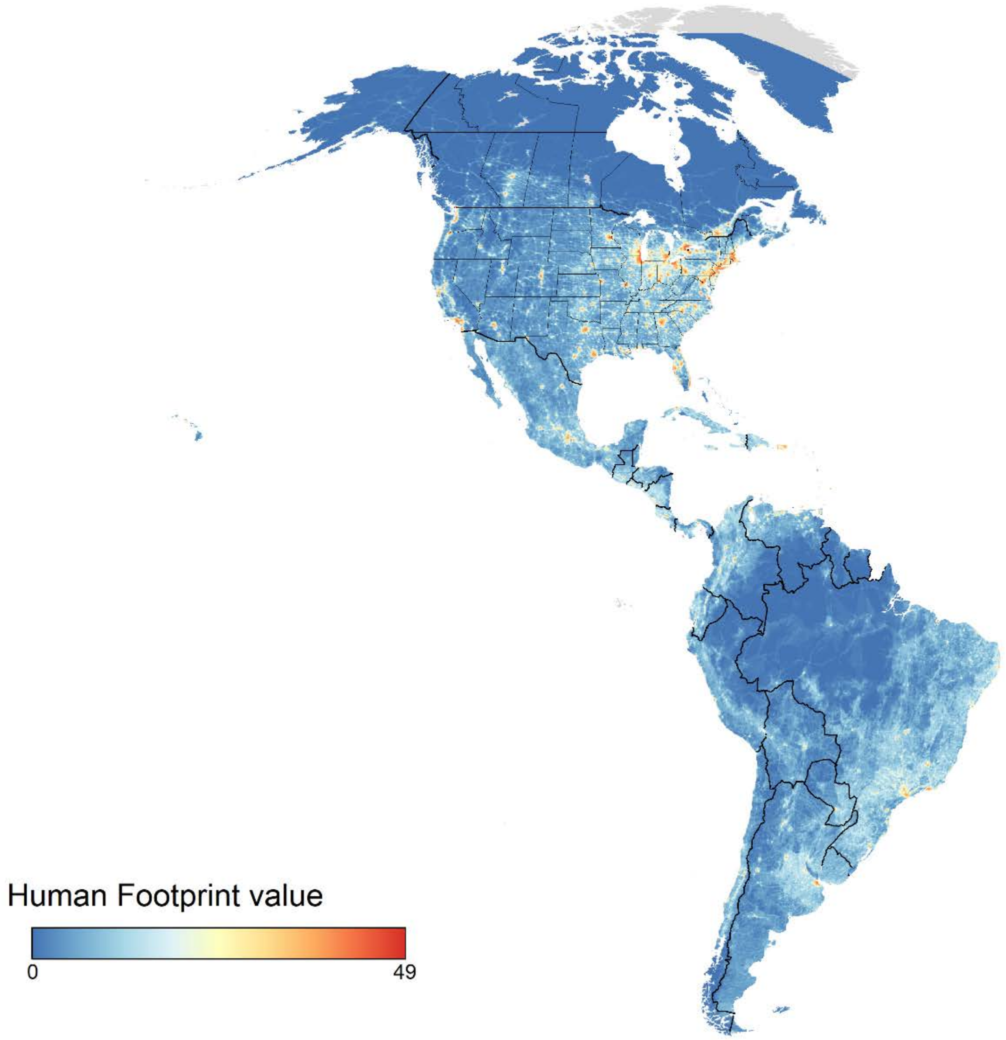

Supplementary Figure 1. Human footprint estimated in 2009. Based on: Venter, O. et al. Sixteen years of change in the global terrestrial human footprint and implications for biodiversity conservation. Nat. Commun. 7:12558 doi: 10.1038/ncomms12558 (2016). Downloaded from: https://datadryad.org/resource/doi:10.5061/dryad.052q5

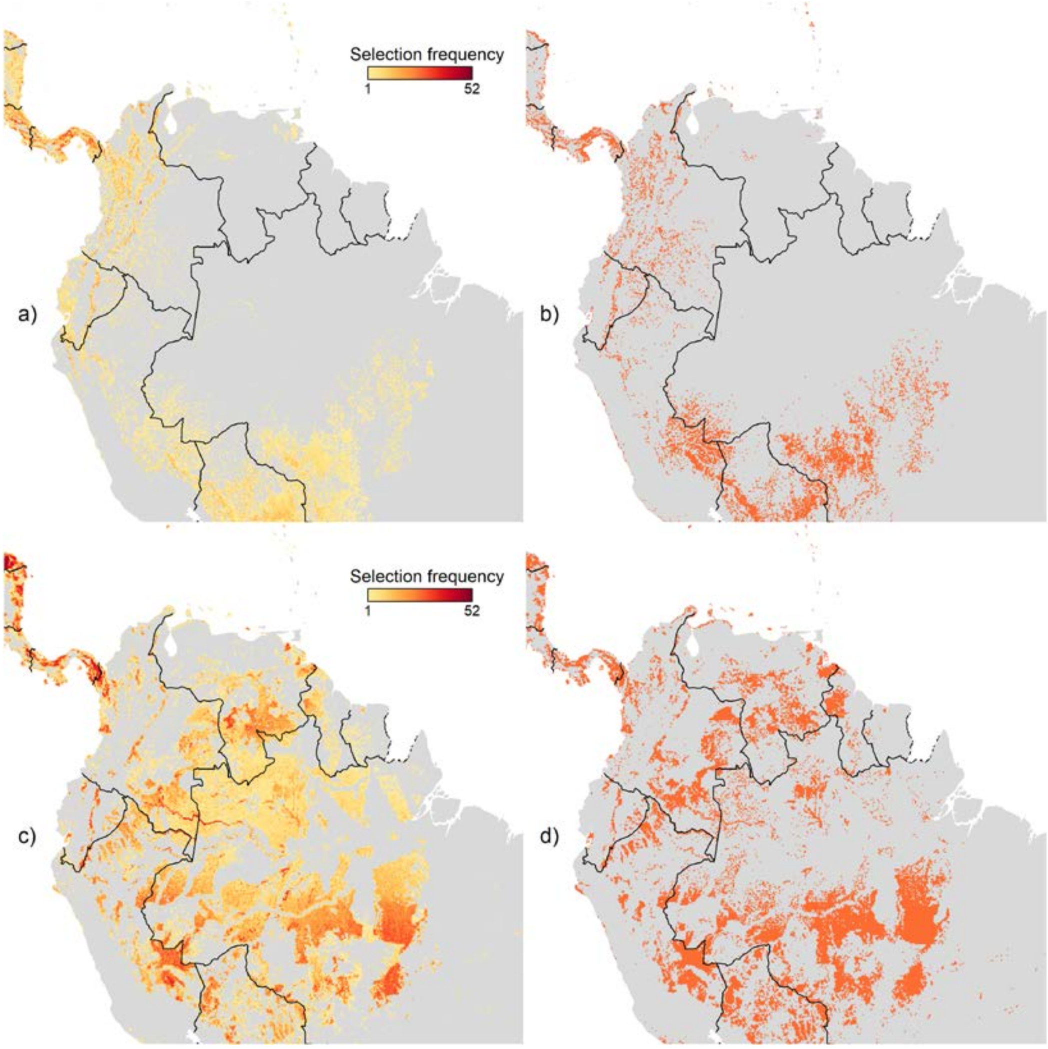

Supplementary Figure 2. Detailed version of Figure 1, focusing on northern South America. Comparison of areas prioritized for weekly and yearly planning under a shared-use approach allowing for the inclusion of human dominated landscapes versus an intact habitat approach that excludes areas of high human footprint. The prioritization is based on a target of 30% of global populations of 117 species of Neotropical migratory birds when each species range is considered as a single population. a) = shared-use, weekly, b) = shared-use, yearly, c) = intact habitat, weekly, d) = intact habitat, yearly.

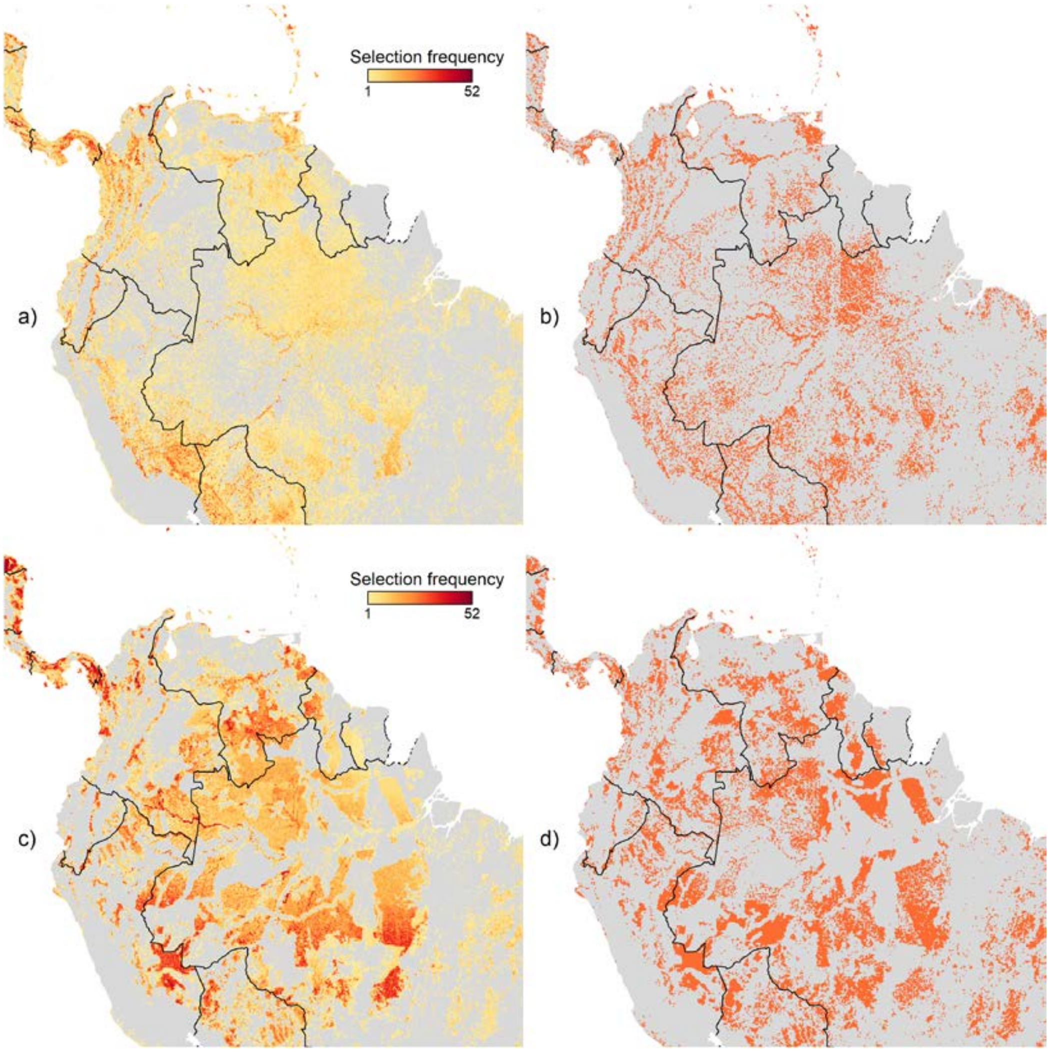

Supplementary Figure 3. Detailed version of Figure 2, focusing on northern South America. Comparison of areas prioritized for weekly and yearly planning under a shared-use approach allowing for the inclusion of human dominated landscapes versus an intact habitat approach that excludes areas of high human footprint. The prioritization is based on a target of 30% of global populations of 117 species of Neotropical migratory birds when each species range is considered with population structure (five regional clusters). a) = shared-use, weekly, b) = shared-use, yearly, c) = intact habitat, weekly, d) = intact habitat, yearly.

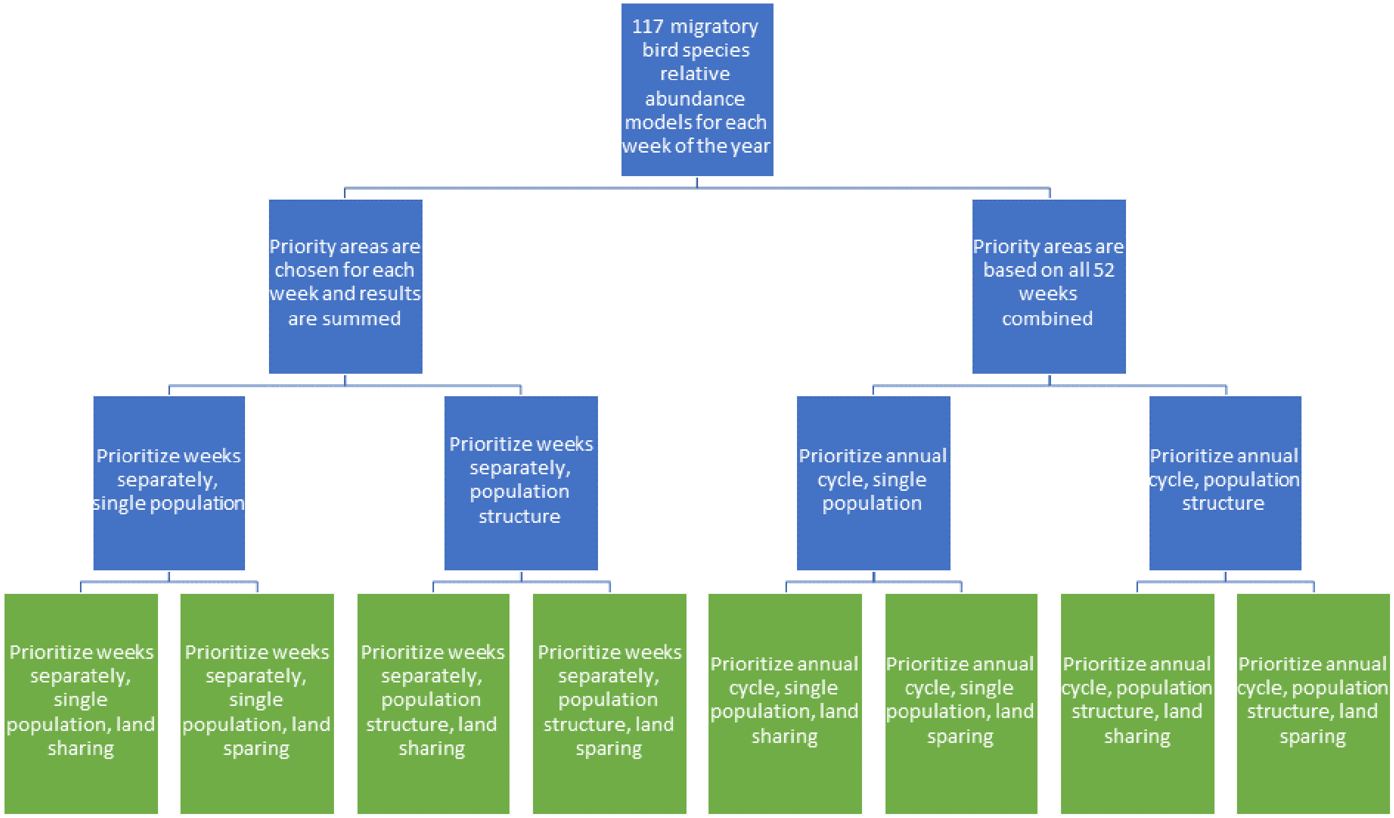

Supplementary Figure 4. Schematic of conservation prioritization scenarios investigated. Runs represents the number of prioritizations we completed per scenario. Boxes in green show the actual scenarios investigated in this study.

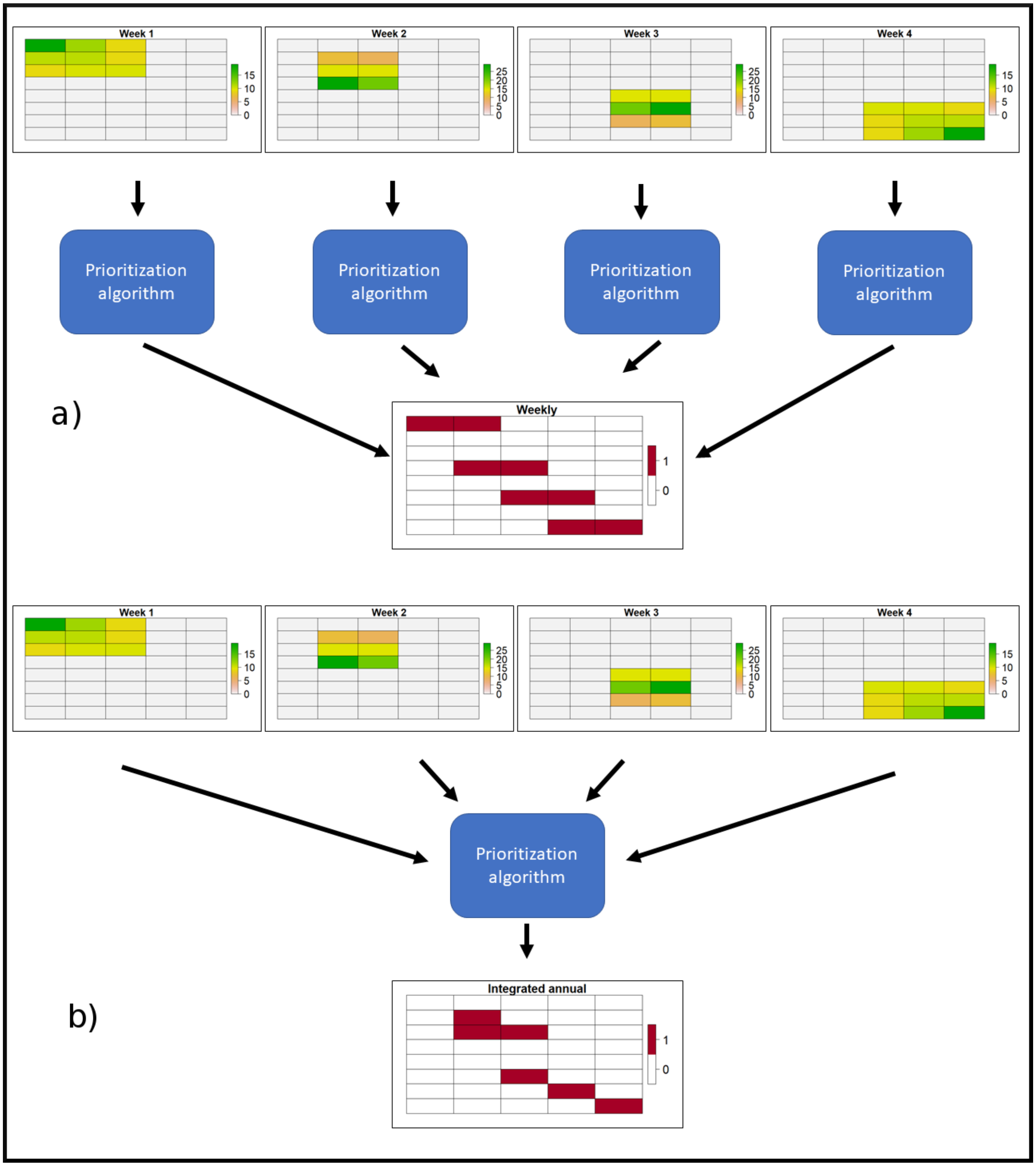

Supplementary Figure 5. Schematic illustration depicting the differences between the weekly and 'integrated annual' approach we used. The example distribution data in the four top panels of a) and b) shows the species abundance distribution for a theoretical species over four weeks. The darker the green the more individuals are present. In the weekly approach a) each week is run through the spatial prioritization approach individually, resulting in a solution for each week.

The bottom figure in a) shows those four maps summed together, highlighting the areas of highest abundance (dark green) for each individual week. In the integrated annual approach b) the distributions from the four weeks enter one spatial prioritization approach at the same time, but as distinct features, meaning they are not combined (e.g. summed) in any way before the prioritization is run. The resulting figure (bottom of b)) shows a gain in area efficiency (i.e. requires less area to meet the 30% target used in both approaches), but this comes at the cost of not capturing areas of highest abundance (dark green) for three out of four weeks (week 1-3). The reason why the 'integrated annual' approach is not capturing the areas of highest abundance for weeks 1-3 is that the algorithm is trying to maximize the co-benefits for all features (i.e. weeks), while minimizing cost, an approach termed complementarity. As a result, the solution does not focus on areas of high abundance alone (as the single feature approaches in a) did), but instead identifies areas that can be selected, which would be beneficial for multiple features/weeks at the same time. As an example, the 30% target for week 1 is covered by the 3 top left cells in the bottom figure of b, but this does not capture the areas of highest abundance for a (moving) species, but might get the leading or trailing edge in some cases because of the integration with other weeks, as shown here. This relatively simple example, using only 40 planning units per week, shows that realized co-benefits between features can look very different from approaches that try to maximize the benefit for a single feature/week.

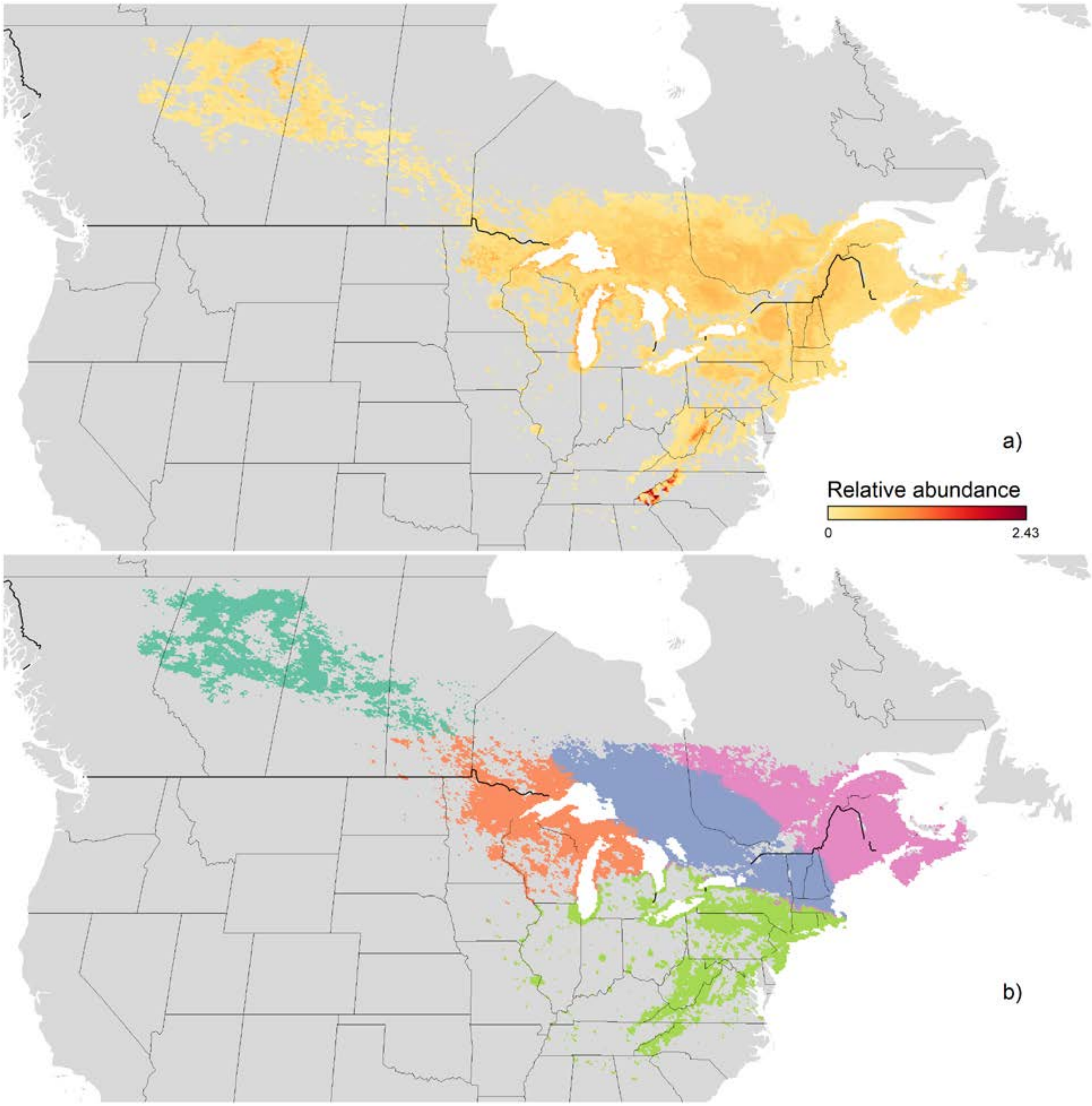

Supplementary Figure 6. Example outcome of clustering approach. For this example, we used Canada Warbler as the species and week of June 6. Figure a) shows the relative abundance of that species in that week of the year, and b) shows the output from the clustering analysis. Each color in b) corresponds to once cluster that was produced using the CLARA algorithm, which is an extension of the k-medoids technique for clustering of large datasets. Each of the clusters in b) was treated as a separate feature (using relative abundance values from a) in scenarios where clustered datasets were used.
