# Supplementary material for: Optimizing the conservation of migratory species over their full annual cycle
Source: Nat Commun. 2019 Apr 15;10:1754. doi: 10.1038/s41467-019-09723-8 (PMC6465267; doi:10.1038/s41467-019-09723-8)
Supplement: Supplementary file 3 — Reporting Summary [file 41467_2019_9723_MOESM3_ESM.pdf]

nature research

Corresponding author(s): Richard Schuster

Last updated by author(s): Mar 14, 2019

Reporting Summary

Nature Research wishes to improve the reproducibility of the work that we publish. This form provides structure for consistency and transparency in reporting. For further information on Nature Research policies, see Authors & Referees and the Editorial Policy Checklist.

Statistics

For all statistical analyses, confirm that the following items are present in the figure legend, table legend, main text, or Methods section.

n/a	Confirmed	
☒	☐	The exact sample size (n) for each experimental group/condition, given as a discrete number and unit of measurement
☒	☐	A statement on whether measurements were taken from distinct samples or whether the same sample was measured repeatedly
☐	☒	The statistical test(s) used AND whether they are one- or two-sided<br>*Only common tests should be described solely by name; describe more complex techniques in the Methods section.*
☒	☐	A description of all covariates tested
☐	☒	A description of any assumptions or corrections, such as tests of normality and adjustment for multiple comparisons
☐	☒	A full description of the statistical parameters including central tendency (e.g. means) or other basic estimates (e.g. regression coefficient) AND variation (e.g. standard deviation) or associated estimates of uncertainty (e.g. confidence intervals)
☒	☐	For null hypothesis testing, the test statistic (e.g. F, t, r) with confidence intervals, effect sizes, degrees of freedom and P value noted<br>*Give P values as exact values whenever suitable.*
☒	☐	For Bayesian analysis, information on the choice of priors and Markov chain Monte Carlo settings
☐	☒	For hierarchical and complex designs, identification of the appropriate level for tests and full reporting of outcomes
☒	☐	Estimates of effect sizes (e.g. Cohen's d, Pearson's r), indicating how they were calculated

Our web collection on statistics for biologists contains articles on many of the points above.

Software and code

Policy information about availability of computer code

Data collection	The eBird citizen science online platform (https://ebird.org/home) was used to collect the data, that citizen scientists collected.
Data analysis	The main software package used to analyze the data in this study was R v3.5.1 (https://cran.r-project.org/). For prioritization analyses we used the R package prioritizr v4.0.2 (https://prioritizr.net), which the corresponding author of this paper is a contributor of. The prioritzr package interfaces with the Gurobi optimization software (http://www.gurobi.com/index). All data and analysis scripts are available here: https://osf.io/58hgs/

For manuscripts utilizing custom algorithms or software that are central to the research but not yet described in published literature, software must be made available to editors/reviewers. We strongly encourage code deposition in a community repository (e.g. GitHub). See the Nature Research guidelines for submitting code & software for further information.

Data

Policy information about availability of data

All manuscripts must include a data availability statement. This statement should provide the following information, where applicable:

- Accession codes, unique identifiers, or web links for publicly available datasets
- A list of figures that have associated raw data
- A description of any restrictions on data availability

All data, computer code used in analysis, files generated from the analysis and outputs such as figures and tables have been deposited and are publicly available here: https://osf.io/58hgs/

Field-specific reporting

Please select the one below that is the best fit for your research. If you are not sure, read the appropriate sections before making your selection.

☐ Life sciences ☐ Behavioural & social sciences ☒ Ecological, evolutionary & environmental sciences

For a reference copy of the document with all sections, see nature.com/documents/nr-reporting-summary-flat.pdf

Ecological, evolutionary & environmental sciences study design

All studies must disclose on these points even when the disclosure is negative.

Study description	We used spatiotemporal exploratory models (STEM) to generate estimates of relative abundance for each species. STEM is a type of species distribution model created as an ensemble of local regression models generated from a spatiotemporal block subsampling design.
Research sample	The bird observation data used to implement STEM came from the eBird citizen-science database. The data included species counts from complete checklists collected under the “traveling”, “stationary”, and “areal” protocols from January 1, 2004 to December 31, 2016 within the spatial extent bounded by 180° to 30° W Longitude (as well as Alaska between 150° E and 180° E). This resulted in a dataset consisting of 14 million checklists collected at 1.7 million unique locations, of which 10% were withheld for model validation.
Sampling strategy	Within each base model, species’ occupancy and abundance was assumed to be stationary. We fit zero-inflated boosted regression trees to predict the observed counts (abundance) of species based on three general classes of predictors: i) spatial predictors to account for spatial (and spatiotemporal) patterns; ii) temporal predictors to account for trends; and iii) predictors that describe the observation/detection process, which account for variation in detection rates, a nuisance when making inference about species occupancy and abundance.
Data collection	The bird observation data used to implement STEM came from the eBird citizen-science database. The data included species counts from complete checklists collected under the “traveling”, “stationary”, and “areal” protocols from January 1, 2004 to December 31, 2016 within the spatial extent bounded by 180° to 30° W Longitude (as well as Alaska between 150° E and 180° E). This resulted in a dataset consisting of 14 million checklists collected at 1.7 million unique locations, of which 10% were withheld for model validation.
Timing and spatial scale	The data included species counts from complete checklists collected under the “traveling”, “stationary”, and “areal” protocols from January 1, 2004 to December 31, 2016 within the spatial extent bounded by 180° to 30° W Longitude (as well as Alaska between 150° E and 180° E).
Data exclusions	No data were excluded from the analysis.
Reproducibility	All data, computer code used in analysis, files generated from the analysis and outputs such as figures and tables have been deposited and are publicly available here: https://osf.io/58hgs/
Randomization	Repeatedly subsampling and partitioning the study extent into grids of spatiotemporal blocks, and then fitting independent regression models (base models) in each block produces an ensemble of partially overlapping local models. Estimates at a given location and date are made by averaging across all the local models that contain the location and date. Combining estimates across the ensemble controls for inter-model variability and adapts to non-stationary predictor–response relationships. To account for spatial variation in the density of the bird observation data, smaller spatiotemporal blocks (10° × 10° × 30 continuous days) were used north of 12° latitude and larger blocks (20° × 20° × 30 continuous days) were used in the southern portion of the study extent.
Blinding	Blinding was not necessary for this study, as it was a model based study on the relative abundance of bird species and subsequent spatial prioritization.

Did the study involve field work? ☐ Yes ☒ No

Reporting for specific materials, systems and methods

We require information from authors about some types of materials, experimental systems and methods used in many studies. Here, indicate whether each material, system or method listed is relevant to your study. If you are not sure if a list item applies to your research, read the appropriate section before selecting a response.

Materials & experimental systems

n/a	Involved in the study
☒	☐ Antibodies
☒	☐ Eukaryotic cell lines
☒	☐ Palaeontology
☐	☒ Animals and other organisms
☒	☐ Human research participants
☒	☐ Clinical data

Methods

n/a	Involved in the study
☒	☐ ChIP-seq
☒	☐ Flow cytometry
☒	☐ MRI-based neuroimaging

Animals and other organisms

Policy information about studies involving animals; ARRIVE guidelines recommended for reporting animal research

Laboratory animals	The study did not involve laboratory animals.
Wild animals	Wild animals were not captured for this study. All data was collected doing passive sampling (observing birds). A species list of all 117 bird species investigated is available in Supplemental Information Table 1.
Field-collected samples	The bird observation data used to implement STEM came from the eBird citizen-science database. The data included species counts from complete checklists collected under the “traveling”, “stationary”, and “areal” protocols from January 1, 2004 to December 31, 2016 within the spatial extent bounded by 180° to 30° W Longitude (as well as Alaska between 150° E and 180° E). This resulted in a dataset consisting of 14 million checklists collected at 1.7 million unique locations, of which 10% were withheld for model validation.
Ethics oversight	No ethical approval was required for this study, as we based our analysis on citizen-science data.

Note that full information on the approval of the study protocol must also be provided in the manuscript.
