# Supplementary material for: Optimizing the conservation of migratory species over their full annual cycle
Source: Nat Commun. 2019 Apr 15;10:1754. doi: 10.1038/s41467-019-09723-8 (PMC6465267; doi:10.1038/s41467-019-09723-8)
Supplement: Supplementary file 4 — Description of Additional Supplementary Files [file 41467_2019_9723_MOESM4_ESM.pdf]

Description of Additional Supplementary Files

File Name: Supplementary Data 1
Description: Supplementary Data 1. The 117 species of Neotropical migratory passerines used in analysis, their range category, as well as PIF score and status. See Methods for range category descriptions. PIF score is the Partners-in-Flight conservation assessment score based on population size, population trend, breeding and non-breeding range size, and threats on the breeding and non-breeding grounds. Higher values indicate greater conservation concern. PIF status indicates species of continental concern with R = red watch list, Y = yellow watch list, T = common bird in steep decline. See Rosenberg et al. (2016) for further detail.

Rosenberg et al. (2016) Partners in Flight Landbird Conservation Plan: 2016 Revision for Canada and Continental United States. Partners in Flight Science Committee. 119 pp.

File Name: Supplementary Movie 1
Description: Species diversity based on relative abundance spatial temporal models of 117 migratory songbird species is shown for every week of the year.
